# Cost-effectiveness of a complex intervention to reduce children’s exposure to second-hand smoke in the home

**DOI:** 10.1186/s12889-018-6140-z

**Published:** 2018-11-13

**Authors:** Charlotte Renwick, Qi Wu, Magdalena Opazo Breton, Rebecca Thorley, John Britton, Sarah Lewis, Elena Ratschen, Steve Parrott

**Affiliations:** 1 Department of Health Sciences, University of York, ARRC, Area 4, Heslington, York, YO10 5DD UK; 2 University of Nottingham, UK Centre for Tobacco and Alcohol Studies, Division of Epidemiology and Public Health, Clinical Sciences Building, City Hospital, Nottingham, NG5 1PB UK; 3 University of Nottingham, Faculty of Medicine & Health Sciences, Division of Epidemiology and Public Health, City Hospital, Nottingham, NG5 1PB UK; 4 University of Nottingham, UK Centre for Tobacco Control Studies, Division of Epidemiology and Public Health, City Hospital, Nottingham, NG5 1PB UK; 5 Department of Health Sciences, Seebohm Rowntree, University of York, Heslington, York, YO10 5DD UK

**Keywords:** Second-hand smoke, Smoking cessation, Passive smoking, Environmental tobacco smoke pollution, Cost-effectiveness analysis

## Abstract

**Background:**

Second-hand smoke (SHS) causes numerous health problems in children such as asthma, respiratory tract infections and sudden infant death syndrome. The home is the main source of exposure to SHS for children, particularly for young children. We estimated the cost-effectiveness of a complex intervention designed to reduce SHS exposure of children whose primary caregiver feels unable or unwilling to quit smoking.

**Methods:**

A cost-effectiveness analysis was carried out alongside an open-label, parallel, randomised controlled trial in deprived communities in Nottingham, England. A complex intervention combining behavioural support, nicotine replacement therapy and personalised feedback on home air quality was compared with usual care. A total number of 205 households were recruited, where the main caregivers were aged 18 and over, with a child aged under five years living in their household reporting smoking inside their home. Analyses for this study were undertaken from the National Health Service/Personal Social Services perspective. All costs were estimated in UK pounds (£) at 2013/14 prices. The primary outcome was the incremental cost-effectiveness of change in air quality in the home, measured as average 16–24 h levels of particulate matter of < 2.5 μm diameter (PM_2.5_), between baseline and 12 weeks. Secondary outcomes included incremental cost per quitter, quit attempts and cigarette consumption in the home. A non-parametric bootstrap re-sampling technique was employed to explore uncertainty around the calculated incremental cost-effectiveness ratios.

**Results:**

The complex intervention achieved reduced PM_2.5_ by 21.6 μg/m^3^ (95% CI: 5.4 to 37.9), with an incremental cost of £283 (95% CI: £254–£313), relative to usual care. The incremental cost-effectiveness ratio was £131 (bootstrapped 95% CI: £72–£467) per additional 10μg/m^3^ reduction in PM_2.5_, or £71 (bootstrapped 95% CI: -£57-£309) per additional quitter.

**Conclusions:**

This trial targeted a socio-economically disadvantaged population that has been neglected within the literature. The complex intervention was more costly but more effective in reducing PM2.5 compared with the usual care. It offers huge potential to reduce children’s’ tobacco-related harm by reducing exposure to SHS in the home. The intervention is considered cost-effective if the decision maker is willing to pay £131 per additional 10μg/m^3^ of PM_2.5_ reduction.

**Trial registration:**

The Smoke Free Homes trial was registered with isrctn.com on 29 January 2013 with the identifier ISRCTN81701383.

## Background

The harmful health effects of second-hand smoke (SHS), also known as environmental tobacco smoke, on children are well established [1, 2]. SHS exposure in children is associated with higher risks of various diseases, including asthma and wheeze [3], respiratory tract infections [4], middle ear disease [5], and even sudden infant death syndrome [2]. The home is the main source of exposure to SHS for children, particularly for young children [6]. It is estimated that around 2 million children are regularly exposed to SHS in the home in the UK [7]. As smoking prevalence is generally higher among caregivers from socio-economically disadvantaged groups [8], children from those households face higher exposure to SHS and increased risk of developing SHS-related diseases [9], which can lead to future health inequalities through intergenerational perpetuation of tobacco dependence and harm [10]. In the UK, SHS smoke in children accounts for 165,000 new episodes of diseases, at an estimated cost of about £23.3 million each year [2]. The long-term costs of treating smoking-caused diseases for smokers who take up smoking as a consequence of exposure to SHS has been estimated at £5.7million per year, plus an additional annual £5.6 million in lost productivity [2]. All these costs are potentially avoidable [2]. In addition to improved child health, reducing air pollution in the home will also benefit other family members.

Smoking cessation programmes are one of the most cost-effective healthcare interventions available in the UK [11–13]. The majority of smoking cessation interventions are focused on people who are motivated to quit; less attention has been paid to those unwilling to quit. This population, although unwilling to quit, may be amenable to stop smoking within the home, reducing the adverse effects on their children through SHS exposure [14]. Despite the rapidly declining smoking prevalence in the UK, it is important to engage smokers from disadvantaged groups and smokers unwilling to quit, who have yet to respond to existing stimuli to quit [15]. A meta-analysis by Rosen et al. [16] evaluated seven studies (six in the US and one in Scotland) aimed at reducing SHS exposure. The results suggested that interventions aimed at reducing SHS exposure, with the primary outcome as air pollution, were effective but limited. However, the cost-effectiveness of these interventions was unclear since no analysis of cost-effectiveness was conducted and no costs of intervention reported.

In this study, we report a cost-effectiveness analysis (CEA) conducted in the context of a randomised controlled trial (RCT) comparing a complex intervention with usual care in reducing children’s SHS exposure in the home [17]. The intervention consisted of both pharmacological and behavioural support as well as a personalised indoor air quality feedback. Our objectives were to compare the costs associated with the complex intervention strategies and the usual care, estimate the effectiveness measured using PM_2.5_ levels, consumption of cigarettes in the home, quit attempts and quit rates and assess the cost-effectiveness of the complex intervention compared with the usual care.

## Methods

### The smoke free homes trial

The trial for which the economic evaluation was conducted was the Smoke Free Homes Trial (Trial registration: ISRCTN81701383), as reported in detail elsewhere [17]. In brief, the trial was an open-label, parallel, RCT based in deprived communities in Nottingham City and County in England. Caregivers aged 18 and over, with a child aged under five living in their household, reported smoking tobacco inside their home and were not willing to quit were recruited and randomised to receive either the complex intervention or usual care. Participants were recruited from 81 English ‘Sure Start’ Children’s Centres across Nottinghamshire. A researcher and a smoke-free homes advisor (SFHA) collected data during home visits at baseline, seven and 12 weeks.

The complex intervention had several components, including behavioural support from a SFHA on how to create a SFH, feedback on the air quality measured in the home, and nicotine replacement therapy (NRT) for temporary abstinence or for reducing number of cigarettes smoked in the home. Participants in the control group received the usual care: a ‘SFH resource pack’ developed by the local Stop Smoking Service. Full details of the study design and intervention have been described in a companion paper presenting the clinical results of the Smoke Free Homes trial [17]. This paper presents a CEA carried out alongside the Smoke Free Homes Trial to assess the value for money of the intervention.

### Resource use

A micro-costing exercise was conducted following the methods of technology appraisal recommended by the National Institute for Health and Care Excellence (NICE) [18]. The main costing component for the alternative strategies was the costs of inputs for the interventions. No wider health care resource use was collected and all trial-related research costs were excluded. Costs for the intervention group were based on three components: (1) up to four one-hour sessions of behavioural support in the home from a SFHA and a minimum of two proactive phone calls or SMS support; (2) NRT and (3) feedback on the air quality (PM_2.5_) of the main living area at baseline, seven and 12 weeks, measured using the Sidepak Aerosol Monitor AM510 (TSI Instruments Ltd., High Wycombe, UK) with the *Trakpro* software already installed. Intervention cost for the usual care group was based on one face-to-face home visit and a resource pack provided by Nottingham Smoke-free homes. Intervention costs, therefore, included the staff cost of the SFHAs along with the relevant travel and telephone expenses, the cost of NRT and the air monitors.

The household cost for contact with the SFHAs was calculated from the treatment log, which recorded the number of visits per household and the length of appointment for baseline, 7 weeks and 12 weeks. Where an appointment time was not given, it was calculated using the estimates of 10 min for graphical feedback and 10 min for behavioural support. For the intervention group, the 24-h visit was estimated at 20 min and the week three visit at 10 min. The advisor wage rate was calculated from the mean of a band 5 and 6 smoking advisor wage [19–21]. Travel time and distance from the hospital were recorded in the treatment log. A return trip was calculated based on the mileage, travel time and advisor wage rate.

Within the treatment log telephone calls were recorded at 10 min per call. Before each visit an additional courtesy call was made to the caregiver. NRT dispensed per person was recorded within the treatment log and costed according to the quantity given per household.

The cost of the air monitor was calculated for 1 year of its 10 life-years (estimated by the manufacturer) and then a cost per use was derived by dividing the annual cost by the number of uses, based on the assumption that the device could be used every other day. Included in the annual cost were the yearly calibration and other fixed costs such as the flow meter. Graphical feedback was costed as 10 min of the appointment time with the associated printing costs.

### Valuation of costs

All resource use was valued in monetary terms, and unit costs were reported in pounds sterling for the financial year 2013/14. All costs were inflated to 2013/14 prices levels where necessary, using the Hospital and Community Health Services pay and price inflation index [22]. The follow-up for the analysis was 12 weeks from randomisation, so no discounting was needed. Table 1 reports the unit costs used in order to cost the intervention. For the support pack, Public Health England provided information on the Smokefree Homes and Cars kit, last distributed in 2012 and the unit cost of £1.45 was given, which was defined as covering production costs only (printing and postage but not fulfilment costs) [23].

**Table 1:** Unit costs and their sources

Resource	Unit cost	Sources
Smoking advisor	£31/h	Smoking Cessation Services (NICE) [19]
Travel	£0.45/mile	Estimated from Smoke Free Homes trial
Telephone call	£0.63/min	Estimated from Smoke Free Homes trial
SMS	£0.04/text	Estimated from Smoke Free Homes trial
Air monitor	£0.60/use	Calculated using manufacturer’s lifetime estimates
Support pack	£1.45/pack	Public Health England (PHE) [23]
Medication (Quantity per pack)		
1.00 mg Nicorette Mouth Spray QuickMist - Double Pack (26)	£19.43	Estimated from Smoke Free Homes trial
1.00 mg Nicorette Mouth Spray QuickMist - Individual Pack (13)	£12.05	
2.00 mg Nicorette Lozenge Nicorette Cool (20)	£4.25	
2.00 mg Nicorette Chewing Gum (30)	£3.41	
2.00 mg Nicorette Chewing Gum Icy White (30)	£3.58	
2.00 mg Nicorette Chewing Gum Icy White (105)	£10.25	
4.00 mg Nicorette Chewing Gum Icy White (105)	£12.05	
15.00 mg Nicorette Inhalator Inhalator (4)	£4.35	
15.00 mg Nicorette Inhalator Inhalator (20)	£15.40	
10.00 mg Nicorette Inhalator (starter pack) (6)	£4.68	
10.00 mg Nicorette Inhalator (refill pack) (42)	£15.39	
1.50 mg Niquitin CQ Lozenge Mint Mini Lozenge (20)	£3.34	
1.50 mg Niquitin CQ Lozenge Mini Lozenge (60)	£9.37	
4.00 mg Niquitin CQ Lozenge Mint Mini Lozenge (20)	£3.18	
4.00 mg Niquitin CQ Lozenge Mini Lozenge (60)	£9.37	
21.00 mg Nicotinell TTS 30 Patch (7)	£8.73	
14.00 mg Nicotinell TTS 20 Patch (7)	£8.24	

### Outcome measures

The primary outcome measure of the trial was the difference in average 16–24 h PM_2.5_ between baseline and 12 weeks. Secondary outcome measures included number of quitters (those who self-reported they had “quit smoking altogether” at 12 weeks), number of quit attempts (lasting longer than 24-h) and difference in cigarette consumption (cigarettes smoked per day in the home) between baseline and 12 weeks.

### Cost-effectiveness analysis

A CEA was undertaken to combine the costs of the trial intervention with PM_2.5_ level and the number of quitters. The primary analysis was conducted on an intent–to-treat (ITT) basis, whereby all randomised households were included and analysed in the groups to which they were randomised. Following NICE guidelines, the analysis was conducted from the NHS/Personal Social Services perspective (including only costs that fall within the healthcare and social services system).

This article’s companion paper used statistical models to adjust for baseline covariates; since there was little difference between those adjusted and those unadjusted, we utilised raw adjustments for our analysis [17]. This allowed us to present all results in the original units (PM2.5, quitters, quit attempts, consumption of cigarettes), which was more meaningful for an economic evaluation than log-transforming PM2.5. The results may differ slightly from the main paper because our multiple imputation model contained cost variables. The incremental cost-effectiveness ratio (ICER), in terms of cost per additional 10μg/m^3^ reduction in PM_2.5_, was calculated using the mean difference in cost between two trial groups divided by the mean difference in effectiveness [24]. The ICER was calculated using 10μg/m^3^ reduction, as this change in PM_2.5_ is utilised by the World Health Organisation (WHO) for mortality risk and therefore considered a meaningful reduction [25]. An additional ICER was calculated for cost per additional quitter. The ICER is calculated using the formula below; ∆ represents difference, E represents effects, C represents the cost of the intervention, while subscripts ‘I’ and ‘UC’ refer to intervention and usual care, respectively [24].

$$ \mathrm{ICER}=\frac{\Delta \mathrm{C}}{\Delta \mathrm{E}} = \frac{{\mathrm{C}}_{\mathrm{I}}-{\mathrm{C}}_{\mathrm{UC}}}{{\mathrm{E}}_{\mathrm{I}}-{\mathrm{E}}_{\mathrm{UC}}} $$

Missing data for outcomes (16% for PM_2.5_) costs (7%) resulted from lost-to-follow-up were imputed using Rubin’s multiple imputation (MI) method [24, 26, 27]. As the data were not normally distributed, we used a non-parametric bootstrap re-sampling method to test the sensitivity of calculated ICERs [28–31]. 5000 estimates of mean costs and mean QALYs were generated for each intervention group and the results were then displayed graphically using a cost-effectiveness plane (CEP) to depict the uncertainty surrounding the mean estimates. To assess the uncertainty surrounding the ICER, bootstrapped 95% confidence intervals (CIs) were generated.

In addition to the primary analysis based on the multiple imputed dataset, a sensitivity analysis was undertaken to repeat the CEA using the 172 out of 204 households who had complete data for the primary outcome and the 188 households who had complete data for number of quitters. All analyses were conducted with Stata version 14.0 and Excel (version 2013). Statistical significance was accepted at *P* < 0.05 in each of the analyses.

## Results

A total number of 205 households were recruited to the trial, but one withdrew from the intervention group, resulting in 204 households (102 in each group) included in the analysis. The majority of primary carers recruited to the trial were female, with just 9% male; the mean age was 28, and 94% were white-British. Full details of trial participants and clinical outcomes are given elsewhere [17].

Table 2 presents a breakdown of the mean cost per household for each element of the intervention. The intervention group had a greater mean total intervention cost than the usual care group (£328 (SD = £151) compared to £45 (SD = £20)). The biggest drivers in this difference were the use of NRT, travel cost and staff time also categorised as feedback time. Greater travel cost was attributable to the extra visits required for the intervention group, since the air monitor drop off/picks ups and the week seven and week 12 follow ups were not included in the costing of the usual care group, as these were considered research costs only (maximum number of visits costed for the intervention group was seven compared to one for the usual care group).

**Table 2:** Mean total cost per household

	Intervention (SD) (*n* = 102)	Usual Care (SD) (*n* = 102)	Difference (95% CI)
Staff	£29 (£8)	£11 (£3)	£18 (£16 to £19)
Feedback	£15 (£2.70)	–	£15 (£14.65 to £15.70)
Telephone	£10 (£4)	£0.10 (£0)	£10 (£8.80 to £10.40)
Travel	£216 (£143)	£32 (£20)	£184 (£156 to £213)
NRT	£56 (£47)	–	£56 (£47 to £65)
Air monitor	£1.68 (£0.32)	–	£1.68 (£1.62 to £1.74)
Support pack	–	£1.45 (£0)	£1.45 (£1.45 to £1.45)
Total	£328 (£151)	£45 (£20)	£283* (£254 to £313)

*Statistically significant (*p*-value< 0.001)

Table 3 reports the base-case results with a decrease of 22.1μg/m^3^ in average 16–24 h PM_2.5_ in the intervention group, compared to just 0.5μg/m^3^ for the usual care group (this is also presented in Table 3 by 10μg/m^3^ decrease, as was used for the ICER)_._ This translated into a 41% mean reduction in the average 16–24-h average PM_2.5_ between baseline and 12 weeks for the intervention group, compared with a 1% mean reduction in the usual care group. The quit rate was higher in the intervention group versus the usual care group (8.0% compared to 4.3%, *p*-value = 0.2614), but did not reach statistical significance in either analysis. Table 4 shows the intervention group experienced a mean reduction of 11 (SD = 10.7) cigarettes smoked in the home per day compared with the usual care’s reduction of 4 (SD = 10.8) fewer cigarettes smoked (*p*-value< 0.001). The quit attempt rate was significantly higher in the intervention group (29.4% compared to 8.6%, *p*-value< 0.001).

**Table 3:** Results of PM_2.5_and quit rate

	Base-case analysis (with imputed data)				Complete case analysis			
	Intervention	Usual Care	Difference (95% CI)	*P*-value	Intervention	Usual care	Difference (95% CI)	*P*-value
No. of households	*n* = 102	*n* = 102	*n* = 204		*n* = 90	*n* = 82	*n* = 172	
Cost (SD)	£328 (£151)	£45 (£20)	£283 (£254 to £313)	< 0.001	£331 (£149)	£46 (£21)	£285 (£252 to £318)	< 0.001
Reduction in PM_2.5_ (ug/m^3^) (SD)	22.1 (65.2)	0.5 (52.0)	21.6 (5.4 to 37.9)	0.0096	24.0 (58.9)	0.9 (52.4)	23.2(6.3 to 40.0)	0.007
Reduction in PM_2.5_ (10μg/m^3^) (SD)	2.21 (6.52)	0.05 (5.20)	2.16 (0.54 to 3.79)	0.0096	2.40 (5.89)	0.09 (5.24)	2.32 (0.63 to 4.0)	0.007
ICER (bootstrapped 95% CI)	£131 (£72 to £467)				£121 (£70 to £471)			
No. of households	*n* = 102	*n* = 102	n = 204		*n* = 95	*n* = 93	*n* = 188	
Cost (SD)	£328 (£151)	£45 (£20)	£283 (£254 to £313)	< 0.001	£331 (£148)	£44 (£21)	£286 (£256 to £317)	< 0.001
Quit rate (%)(SD)	8.0% (27.0%)	4.3% (19.6%)	3.7% (−2.8% to 10.2%)	0.2614	8.4% (27.9%)	4.3% (20.4%)	4.1% (−2.9% to 11.2%)	0.248
ICER (bootstrapped 95% CI)	£71 (−£57 to £309)				£72 (−£22 to £313)

**Table 4:** Results of consumption of cigarettes in the home and quit attempts

	Base-case analysis (with imputed data)				Complete case analysis			
	Intervention	Usual Care	Difference (95% CI)	*P*-value	Intervention	Usual care	Difference (95% CI)	*P*-value
No. of households	*n* = 102	*n* = 102	*n* = 204		*n* = 95	*n* = 93	*n* = 188	
Cost (SD)	£328 (£151)	£45 (£20)	£283 (£254 to £313)	< 0.001	£330(£148)	£44(£21)	£286 (£256 to £317)	< 0.001
Reduction of consumption in the home (no. of cigarettes per day) (SD)	11 (10.7)	4 (10.8)	7 (9.8 to 3.9 to 9.8)	< 0.001	11 (10.8)	4 (10.5)	7.5 (4.4 to 10.6)	< 0.001
No. of households	*n* = 102	*n* = 102	*n* = 204		*n* = 93	*n* = 93	*n* = 186	
Cost (SD)	£328 (£151)	£45 (£20)	£283 (£254 to £313)	< 0.001	£331 (£149)	£44 (£21)	£286 (£256 to £318))	< 0.001
Quit attempt rate (%)(SD)	29.4% (43.6%)	8.6% (27.1%)	20.7% (10.8% to 30.8%)	< 0.001	29% (45.6%)	8.6% (28.2%)	20.4% (7.3% to 32.0%)	< 0.001

Tables 3 and 4 report the results of the complete case analysis, although there was little variation. The results only differed slightly between the base-case analysis and the complete case analysis with the average 16–24 h PM_2.5_ results generally better for the intervention group in the base-case.

The primary outcome, average 16 to 24-h PM2.5, was selected for the CEA along with quitters. Table 3 presents the ICERs which combine the differential costs of the two groups with the differential outcome measures. The intervention group was more costly than the usual care group, but had a greater decrease in the PM2.5 level. This resulted in an ICER of £131 (bootstrapped 95% CI: £72–£467) per additional 10μg/m3 reduction of 16 to 24-h PM2.5. Analyses of the quitters resulted in an ICER of £71 (−£57 to £309) per additional quitter. The uncertainty surrounding this ICER was reflected by the bootstrapped CIs for both analyses. The complete case analysis showed very similar results.

**Fig. 1:**
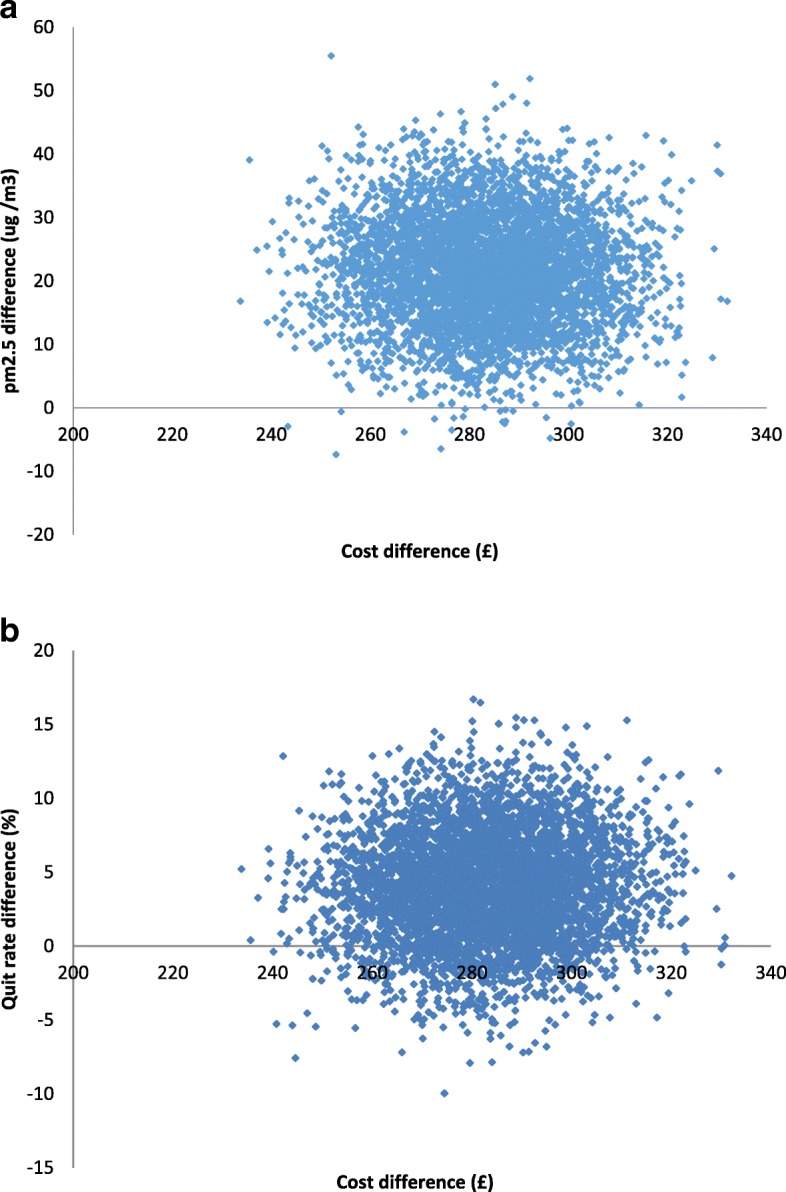
The bootstrapping results of the 5000 re-samples for each outcome: **a**. PM2.5; **b**. Quit rate.

The bootstrapping results of the 5000 re-samples for each outcome were plotted on a CEP (Fig. 1), visually displaying any uncertainty surrounding the mean differences in costs and benefits between the intervention and usual care groups. Figure 1ashows this uncertainty for the primary outcome (PM2.5 difference). The majority of the plots fall in the south-east quadrant, this indicates although the intervention is always more costly, it is more likely to be effective at reducing PM_2.5_ levels, compared with usual care. The quit rate is more uncertain as shown by some of the plots falling in the south-east region of the CEP (Fig. 1b) therefore there is a lack of evidence to show the intervention was more effective at helping people to quit. This is unsurprising, as this was not the main aim of the trial.

## Discussion

To our knowledge, the present study is the first full economic evaluation alongside an RCT to assess the cost-effectiveness of a complex intervention designed to reduce children’s exposure to SHS in the home. The study has shown the complex intervention significantly reduced SHS exposure in the home among families in which parents had expressed no interest in quitting smoking previously. Decision makers must be willing to pay £131(bootstrapped 95% CI: £72–£467) per additional 10μg/m^3^ reduction of PM_2.5_ in order to reduce SHS in the home and limit harm to children. It was presented per additional 10μg/m as this was seen as a meaningful reduction and is used by WHO when presenting mortality risk [25]. The results revealed the intervention was more costly (mean cost: £328 (SD = £151) vs £45 (SD = £20)) than usual care, but produced better outcomes. Total mean costs were £283 (95% CI: £254 to £313) higher in the intervention group, this was mostly attributable to the cost of travel with a mean difference of £184 (95% CI: £156 to £213) and the cost of NRT with a mean difference of £56 (95% CI: £47 to £65). Based on WHO recommendations, the safe level of PM_2.5_is < 25 μg/m^3^ (24-h mean), however children are recognised as particularly vulnerable and there is no threshold below which adverse health effects do not occur [25, 32]. Neither the usual care nor intervention group met the WHO threshold at 12 weeks (usual care = 47 μg/m^3^, intervention = 32 μg/m^3^), but the intervention group did experience an overall reduction of 41% from baseline to 12 weeks.

The strength of the economic analysis has been impacted by a few limitations of the study. Firstly, wider health care resource use beyond the trial interventions was not collected and this plays an important role in the drive behind reducing SHS exposure. This cost dimension would have strengthened the economic analysis and brought it more in line with NICE guidelines. Secondly, the trial follow-up period was only 12 weeks, and it may not be long enough to capture the full impact of the intervention. Further research with longer-term follow-up is needed to explore any potential long-term benefits from the intervention.

This longer follow-up would also allow the use of the EQ-5D and the subsequent calculation of Quality Adjusted Life-Years (QALYs), a generic health measure [33–35]. QALYs can be used and easily compared across interventions with a willingness to pay thresholds range of £20,000 to £30,000 per additional QALY gained to decide cost-effectiveness [18]. However, QALYs can be insensitive to disease-specific conditions, in particular those concerning mental health [36]. Thirdly, no definitive conclusion about cost-effectiveness can be made due to the absence of decision-making thresholds for any of the outcomes collected alongside the trial.

These limitations aside, this trial targeted a socio-economically disadvantaged population that has been neglected within the literature. Previous research showed great success with sophisticated methods for interventions aimed at smokers who are serious about and willing to quit [37, 38]. Despite the rapidly declining smoking prevalence, it is important to engage with smokers who have not yet responded to existing stimuli to quit [15]. New and innovative approaches are needed to target those who are not willing to quit, but may be willing to reduce consumption in the home, thereby limiting the impact of SHS on children. Our results showed a reduced number of cigarettes being smoked inside the home and lower PM_2.5_ level, indicating some success in the trial aims. Although not statistically significant, this intervention group had a 3.7% higher quit rate than usual care, suggesting even those who are seemingly not willing to quit are still able to and should not be ignored, but this result should be taken with caution due to the high level of uncertainty. The results showed a higher number of quit attempts in the intervention group (20.8% higher quit attempt rate). Chaiton et al. [39] argue when taking into account those smokers who are less willing to quit, it may take 30 or more quit attempts before being successful. Therefore, these increased quit attempts may indicate a likelihood of longer term success.

More high quality research such as larger RCTs with longer follow-up periods, generic health outcome measures and collection of wider healthcare resource use is needed to explore the impact of complex interventions on reducing children’s SHS exposure. Furthermore, studies exploring interventions that help those who are not willing to quit smoking are needed. These interventions may have short term objectives of reduced consumption, but with the potential of long term success of quitting.

## Conclusions

This trial targeted a socio-economically disadvantaged population that has been neglected within the literature. The complex intervention was more costly but more effective in reducing PM2.5 compared with the usual care. It offers huge potential to reduce children’s’ tobacco-related harm by reducing exposure to secondhand tobacco smoke in the home. The intervention is considered cost-effective if the decision maker is willing to pay £131 per additional 10μg/m^3^ of PM_2.5_ reduction.

## Abbreviations

CEA: Cost-effectiveness analysis
CEP: Cost-effectiveness plane
CI: Confidence interval
ICER: Incremental cost-effectiveness ratio
MI: Multiple imputation
NICE: National Institute for Health and Care Excellence
NRT: Nicotine replacement therapy
QALY: Quality adjusted life years
RCT: Randomised controlled trial
SFHA: Smoke free homes advisor
SHS: Second-hand smoke
WHO: World Health Organisation
